# Epilepsy

**Published:** 1899-03

**Authors:** E. Carleton

**Affiliations:** New York City


### EPILEPSY.

*A Case from Practice.*

By E. Carleton, M. D., New York City.

This malady has pestered mankind for ages. It has been persistently studied by the medical profession, and yet is not understood. Anatomical investigation has failed to give any explanation of epilepsy. Every kind of lesion has been discovered; and, again, every particle of tissue in the human frame has been found to be apparently in perfect health. The pathologist surrenders. The disease is styled "functional." The homœopathic physician, alone, understands that in this, as in all other forms of sickness, all the symptoms of the individual constitute the only guide to the similar, curative agent, and that nothing more is needed. The general symptoms have, indeed, been carefully recorded by many able observers, but for diagnosis, classification, and prognosis mainly. Therefore it is no wonder that the prevailing method of treatment of epileptics is scarcely ever anything but disappointing—so much so that the report of a cure by a physician is pretty sure to be assailed by his professional brethren, who think the case was not properly diagnosed. To come within the pale, either epilepsia mitior, sudden, temporary, but absolute arrest of both perception and volition; or of epilepsia gravior—complete loss of consciousness; tonic muscular spasm, more on one side; impeded respiration, dilatation of pupils; later, in addition, clonic convulsion, frothing at the mouth, and finally stupor, must be present.

The prevailing treatment is disappointing. What can Old Physic do, but give a bromide, and that in huge doses, which bring but temporary palliation and respite, and work much damage to the patient? Yes, they can besides perform operations, and thereby palliate some cases.

Formidable as is this disease, it can be made to yield to pure Homœopathy. Bönninghausen is said to have cured thirty cases; and all of us have had a measure of success in the same direction. It seems desirable to place on record, from time to time, some unquestionable cures, and thus establish the truth. The following case is given in illustration, for that reason. I have followed the notes in my case book rather closely, thus making a somewhat longer, but, it is to be hoped, also more natural and interesting history.

Mrs. C———, aged thirty-four, first applied for help January 19th, 1893, and described her case as follows: Seven years before gave birth to a child at the seventh month. Digitalis, given for dropsy of the mother, had killed the child, and miscarriage soon followed. Treatment, heroic Allopathy—curette, Ergot, much Whisky. Six days after delivery she was partially paralyzed by embolism of right, middle meningeal artery, the left limbs gradually becoming useless. Consultations were held by eminent physicians, including a specialist of international reputation in mental and nervous diseases. They gave enormous doses of Iodide of Potassium, Nitroglycerine, and perhaps other drugs. After a long while she became able to walk a little, slowly, dragging the left foot, and to swing the arm and clasp slowly with the fingers. A peculiar feature was her inability to unclasp without aid.

About the time that she became able to limp, epileptic seizures were noticed, gradually becoming more frequent and severe. Patient stated that for a month before their appearance she had, by prescription, had ice applied to the spine, and that it "irritated" her brain. Again the same eminent counsel were brought in. Their diagnosis was true epilepsy. By their direction the attacks were called "fainting spells." She has never been undeceived. Her mother told me, and described the appearance and actions of the patient. The largest permissible doses of bromides in variety, Hypo-phosphites, Ammonia, and Fowler's Solution of Arsenic were given. Gradually the attacks diminished in frequency and violence to a certain point, and refused to budge any farther. Their type remained unchanged. Then the drugs named were stopped. Cascara-sagrada was ordered for constipation, and she took much of that. In fact, all the way through her physicians seemed to study their patient's ability to swallow medicine, and to give as much as they thought she could stand. When a "fit" occurred she was simply kept quiet. Old Physic confessed defeat, and retired.

At our first interview it developed that the attacks were irregular, averaging, say about one in three months, usually preceded, about 11 A. M., by fright, palpitation of heart, dizziness, occasionally nausea; between two and three next morning her mother would hear a scream; find patient unconscious, with head twisted to the right, thumbs clenched by the fingers, tonic and clonic spasms, eyes rolled up, and pupils dilated, chewing and frothing at the mouth, tongue bitten, followed by long, stupid sleep.

She was a woman of medium size, dark, languid, calm, of sweet disposition. Reproof would cause dark areas to appear under the eyes; easily hurt, silent, submissive. Strange lapses of memory, temporary and partial, occurred at uncertain times. Itching of scalp; hair greatly inclined to plica polonica; habitually patted the floor with her right foot; left hand and foot in condition described; cold extremities since childhood; poor appetite, good sleep; menses every six weeks, rather free.

Here was a problem to solve—how best to antidote Digitalis, which had suppressed the dropsy, killed the child, and produced miscarriage; also the currettement, Whisky, and loads of drugs; also the ice to the spine, which had precipitated epileptic attacks; and the monumental drugging that ensued. How best to antidote and to cure; for the condition that led to the first bad drugging must be considered. I stuck to the law of cure, studied the symptoms present, and was by them guided to the remedy. No promises were asked for or given, but hope was expressed by me that her condition should be mitigated. The constitution and mental state pointed unmistakably to Ignatia. The spasmodic symptoms and those related thereto, with the concomitants, were fairly well covered by the same remedy. Although Nux-vomica is oftener the antidote to drugs, still Strychnia, the principal constituent of it, exists also in Ignatia. A foot-note by Hering, page 178, Vol. VI, *Guiding Symptoms*, reads thus: "There are many more Ignatia persons in North America than Nux-vomica persons, at least this side of Mason and Dixon's line. C. Hg." Ignatia was chosen. In my experience a drug, especially a vegetable, which is to act in the double capacity of antidote and curative, is often best given in medium potency, at short intervals, until reaction is observed, and then stop.

Accordingly, Ignatia, 200th, in water, was given every two hours until improvement should be observed.

As expected, constipation was the first item complained of. All drugs, including tea and coffee, had been forbidden. Said the patient: "What shall I do?" My answer was: "Do nothing unless great oppression or uneasiness develops, then use a simple enema of warm water." She was obedient, and constipation steadily improved. My notes do not show when improvement in the case was first noticed, but my recollection is that it was a few weeks—less than a month. I know that improvement soon abated when the medicine was stopped; that it was given again, less frequently and for a shorter period before improvement again appeared; that the second time of improvement was longer than the first, and that the same plan was continued—the need of medicine gradually tapering off. July first, six months after the first study of the case, the following letter was received from the mother:

"She is feeling rather languid. The dizzy turns which seem always to mark the latter part of the interval between the serious attacks, have begun, and she has had two, each one a week apart. They generally begin at four weeks after the serious attack. This time it was six weeks before the first one appeared. Since you have had Mrs. C——— in charge the intervals are longer each time. She is run down and feels poorly."

The next letter was August 14th, three weeks later than the one just read. This is the report:

"She is feeling weak and miserable. Her monthly sickness came August 1st, after the usual six weeks' interval, and ceased the 8th. Yesterday (13th) she was again taken, and has quite a flow. She is very black around the eyes, and looks ill. Appetite and sleep all right. It will be eleven weeks to-morrow since she had one of her bad attacks. A week ago she had an attack of indigestion, which to my great amazement, did not cause a convulsive attack. This is, of course, a great gain."

The next day I sent to Long Branch, where they were, two powders of Ignatia, 200th, one to be taken at bed-time, and the other next morning. They were too late, as this letter, dated August 16th, will show:

"Mrs. C——— had a convulsive attack yesterday, 6 A. M. It was slight—all over in fifteen minutes. So you see there is improvement. The interval (eleven weeks) was the same as before, viz., March 14th, May 30th, August 15th. I forgot to tell you in my last note there is improvement in the left arm. Certain quick movements not made before. The flow still continues very freely."

Improvement then went on with little interruption, each seizure being followed by a dose of Ignatia, 200th. The last attack was July 17th, 1894, as I learn from a long letter from the mother, expressing gratitude, written July 18th, 1895. This extract will show:

"I write with a full heart, for it is just one year since my daughter's last attack. She is well."

A year ago, June, 1897, I made careful examination of her case, and found nothing wrong. Function was restored to extremities; no "fainting fits;" felt well. This June, 1898, I find no fault. Four years having elapsed without any trouble, the patient being in good health, it is safe to say that this severe and complicated case has been cured.

DISCUSSION.

Dr. Kennedy—Epilepsy is a disease which I believe we all dread to meet. I have had but little experience in treating epilepsy. In one or two cases, possibly three, I have been able to prevent the return of the attacks after treating them some time. In the other cases that have come under my observation I have tried the best I could and failed. I do not think that Homœopathy failed, but I did not find the remedy.

Dr. Kimball—The most difficult cases are those in which there has been excessive use of bromides. It seems to make them almost incurable. I have had several and my treatment was nothing more than palliative; they were not cured. As

Dr. Kennedy said, that was not the fault of Homœopathy, but my fault.

Dr. Close—I regard that as a remarkable case beautifully cured. I wish I had in my records a case which would match it, but I am sorry to say that I have not. I have had some very severe and very troublesome cases of epilepsy, and have not perfectly cured any of them. I am inclined to think that one secret of Dr. Carleton's success was his close adherence to the maxim of Davy Crockett:—"Be sure you are right, then go ahead." It is necessary to find at least a close simile, if not the simillimum, and then adhere to it until it accomplishes its work. I am convinced that the steady concentration of the physician's will on the patient in the desire and purpose to cure has something to do with it also. But he must not waver. If he wavers, or doubts, thinking some other remedy may do better than the one selected, he will be very apt to fail.

Dr. Patch—I have been much interested for the last year or two in watching a case which has been under the care of Dr. Ranney, of New York. The case has been the rounds. The people come on to New York and the patient remains under treatment for a few days. So far there has been only slight, if any, diminution in the frequency of the attacks. I think increased care in diet may have had much to do with even that. I often wonder if the cases of epilepsy which come on after the suppression of external disease differ from any other form of the disease. I have one case in mind. A case of eczema in childhood. That was suppressed, and epileptoid symptoms appeared, complicated with intermittent fever. I have been able to bring about a return of the intermittent fever, and hope to be able to cure the entire case in the future.

Dr. Davis—I had, a short time ago, a case which would perhaps hardly be diagnosed as epilepsy, but the cause, so far as we knew, was a blow on the head some time before the seizures commenced. Had them at rate of eight or ten a day. Some of them would last some time, some only a few minutes.

9

Had been under the care of many physicians but with no result. Influenced by the history of the case I gave Arnica 1M. As the patient lived out of town I sent medicine by mail. The report was that the patient was much better. A recent letter said that she had no return of attack thus far.

